# Assessing Neighborhood Characteristics and Their Association with Prenatal Maternal Stress, Depressive Symptoms, and Well-Being in Eight Culturally Diverse Cities: A Cross-Sectional Study

**DOI:** 10.3390/ijerph22030456

**Published:** 2025-03-20

**Authors:** Laura Campo-Tena, Gabriela Diana Roman, Aja Louise Murray, Bao Yen Luong-Thanh, Marguerite Marlow, Yasmeen Anwer, Awurabena Dadzie, Sarah Foley, Sandra Stuart Hernandez, Carene Lindsay, Shobhavi Randeny, Joanne A. Smith, Diana Taut, Manuel P. Eisner

**Affiliations:** 1 Institute of Criminology, University of Cambridge, Cambridge CB3 9DA, UK; 2 Department of Psychology, University of Edinburgh, Edinburgh EH8 9YL, UK; 3 Department of Epidemiology, Biostatistics & Demography, Hue University of Medicine and Pharmacy, Hue City 7720101, Vietnam; 4 Institute for Life Course Health Research, Department of Global Health, Stellenbosch University, Cape Town 7535, South Africa; 5 Global Health Department, Health Services Academy, Islamabad 44000, Pakistan; 6 Department of Psychology, University of Ghana, Accra P.O. Box LG 25, Ghana; 7 Moray House School of Education and Sport, University of Edinburgh, Edinburgh EH8 8AQ, UK; 8 Child Protection Unit, University of the Philippines, Manila 1000, Philippines; 9 Caribbean Institute for Health Research, The University of the West Indies, Kingston 00000, Jamaica; 10 Department of Paediatrics, Faculty of Medicine, University of Kelaniya, Kelaniya 11600, Sri Lanka; 11 Department of Psychology, Babes-Bolyai University, 400347 Cluj-Napoca, Romania

**Keywords:** neighborhood characteristics, maternal stress, depressive symptoms, well-being, validation

## Abstract

Despite growing evidence on the influence of neighborhood characteristics on maternal well-being, there is a critical gap in the availability of validated instruments for measuring these constructs across different cultural settings. Existing neighborhood-related scales often lack cross-cultural validation, limiting their applicability in low- and middle-income countries. Understanding the impact of neighborhood characteristics is crucial given its multigenerational impact. We used data from the Evidence for Better Lives dataset to assess the conceptual and measurement equivalence of the community scales of neighborhood cohesion, intergenerational closure, and neighborhood and social disorder, testing for measurement invariance across eight low- and middle-income countries. Secondly, we examined patterns of associations with prenatal maternal stress, well-being, and depressive symptoms through the use of nomological networks. We found that the conceptual and measurement equivalence of the neighborhood domains were comparable across the eight studied countries. Additionally, our results suggest that higher levels of neighborhood and social disorder and lower levels of cohesion and intergenerational closure in the community are associated with adverse maternal outcomes across the included sites. The results of this study stress the importance of exploring the community context when assessing maternal well-being and supports the need to advocate for community-based interventions that promote safer physical and social environments within maternal programs and urban planning.

## 1. Introduction

Considerable evidence from high-income countries suggests that neighborhood characteristics—such as social disorder, social deprivation, and material deprivation—can have detrimental effects on the mental health and well-being of pregnant women [1,2,3]. Furthermore, it has been well-established that women who live in disadvantaged neighborhoods have higher risk of adverse outcomes during pregnancy and post-birth, including low birthweight, preterm birth, stillbirth, and infant mortality [4,5,6]. Understanding the impact of neighborhood characteristics is therefore important given its multigenerational impact.

Neighborhoods are a social structure that encompasses the immediate setting in which individuals reside and that influences their context or behaviors [7]. This assertion builds on an important contribution by Bronfenbrenner [7], who introduced a bioecological framework to explain children’s health and development and who considered that human development takes place within different nested socially organized environments that influence the development of health and well-being through direct and indirect interactions [8].

The interest in understanding how the community context influences the population’s health has increased in recent years [9,10]. Research has demonstrated that neighborhood-level factors do not operate independently, but interact with individual-level factors, establishing a reciprocal loop [11]. Although the measurement of ecological characteristics of neighborhoods is considerably less established in epidemiology than the measurement of individual-level variables [10], both are important when exploring the influence of social capital [12]. Constructs such as neighborhood trust and cohesion, neighborhood disorder, and the strength of intergenerational closure are particularly relevant constructs for understanding neighborhood effects on maternal and family well-being.

In the broadest sense, neighborhood cohesion is defined as more of a structural concept, involving participation in local organizations, links between social groups, and implication in collective activities [13,14]. Research has suggested that neighborhood cohesion is more than just a condition or social force. Instead, it should be understood as the collective state or inclination of the community—i.e., the sense of residential togetherness that residents experience [15]. Therefore, it relates to the residents’ feeling of belonging, but also to the overall attractiveness of living in a specific area [16]. It should be noted that the highest levels of cohesion are observed in large cities with the greatest density of amenities, while the lowest levels are found in suburban settlements, highlighting their functional shortcomings [16]. The presence of neighborhood cohesion has been identified as a protective factor for anxiety in mothers of young children [17].

A second concept that is important for understanding well-being outcomes from an ecological perspective is intergenerational closure. Intergenerational closure is related to social cohesion and it is based on a multilevel perspective. It refers to the presence of dense networks formed between adults and children in the community [18]. This concept was also described by Coleman as the connections between families of children who are friends, which help strengthen social cohesion within local settings like schools or neighborhoods [19]. Intergenerational closure operates through two key mechanisms. In the “top-down” closure, parents form social ties first, creating opportunities for their children to develop friendships. In contrast, “bottom-up” closure occurs when children form friendships first, prompting their parents to connect through activities like arranging playdates or school pickups. Both processes strengthen social networks and foster community cohesion [20]. Its impact on well-being has not been explored in neighborhood research to the same extent as other dimensions, especially in pregnant women.

A third concept that has been prominent in social environmental research has been neighborhood disorder, which generally comprises observed or perceived physical and social features of neighborhoods that can undermine the quality of life of residents [9,21]. Research on this factor in the perinatal period is very limited, though emerging. For instance, low levels of social disorder in neighborhoods during childhood and pregnancy have been associated with lower levels of postpartum depressive symptoms in African American women [22]. In this context, higher amounts of physical neighborhood disorder have been associated with increased maternal smoking and inadequate weight gain during pregnancy [23]. Additionally, neighborhood deprivation has been found to contribute to an increased risk of severe maternal morbidity [24].

A major concern when exploring neighborhood characteristics is the limited availability of validated instruments [13,25] and the fact that these are rarely validated across different populations, cultures, and languages, hampering the achievement of international surveys and meaningful cross-cultural comparisons [13]. How neighborhoods are organized based on socioeconomic characteristics, social disorder, and exposure to violence is important to assess when comparing culturally diverse settings [26]. In this sense, establishing invariance, that is the equivalence of a concept, is important to support cross-context comparisons that might illuminate which contexts are most in need of interventions and to test hypotheses about the effects of macro-level variables. To this point, there is little evidence for a cross-country validation of neighborhood-related scales and the research that exists focuses on high-income countries [27,28]. However, cross-country comparative design has been previously used to gain insights into important issues such as prenatal attachment [29] and depressive symptoms in pregnant women [30] in low- and middle- income countries (LMICs). There is also limited research on whether social and material features of neighborhoods are similarly associated with constructs of maternal stress, depression, and well-being across societies. Increasing knowledge on the role neighborhoods play in relation to women’s well-being during pregnancy is crucial as it represents a unique window of opportunity that can both benefit women and their offspring.

### The Current Study

First, we assess the conceptual and measurement equivalence of the community domains of neighborhood cohesion, intergenerational closure, and neighborhood and social disorder, testing for measurement invariance across eight LMICs. Our first research question is as follows: To what extent do these neighborhood characteristics demonstrate conceptual and measurement equivalence across culturally diverse sites? We hypothesize that these constructs will exhibit partial measurement invariance, indicating that they can be meaningfully compared across sites while allowing for some cultural variation. Second, we examine patterns of associations with prenatal maternal stress, well-being, and depressive symptoms. Our second research question is as follows: How are neighborhood cohesion, intergenerational closure, and neighborhood and social disorder associated with prenatal maternal stress, well-being, and depressive symptoms across different LMICs? We hypothesize that higher levels of neighborhood and social disorder will be associated with increased maternal stress and depressive symptoms, whereas greater neighborhood cohesion and intergenerational closure will be linked to higher maternal well-being.

This research adds to the limited body of knowledge on the association between community characteristics and health outcomes in pregnant women in LMICs, which may inform community-based interventions in different societies. Validating an instrument assessing neighborhood characteristics across cultures may encourage subsequent important cross-cultural comparisons.

## 2. Materials and Methods

### 2.1. Data

This study analyzed data from the Evidence for Better Lives (EBLS) dataset. The EBLS is a prospective birth cohort study conducted in eight low- and middle-income cities in diverse regions, including Kingston (Jamaica), Koforidua (Ghana), Worcester (South Africa), Cluj-Napoca (Romania), Tarlai Kalan (Pakistan), Ragama (Sri Lanka), Hue (Vietnam), and Valenzuela City (the Philippines). The EBLS was designed to provide high-quality longitudinal evidence to support effective interventions to tackle violence against women and children. The research sites were selected based on several criteria, including economic status (low-middle and upper-middle income), regional diversity, political receptiveness to research, presence of skilled local research teams, and existing connections to prenatal healthcare services. The selection aimed to capture social and cultural diversity, reflected in international indices like homicide rates, gender inequality, Gross Domestic Product (GDP), and birth rates [31]. The EBLS currently consists of three completed waves of data collection; the first wave—the focus of the current study—was conducted when participating women were in the third trimester of pregnancy.

The EBLS questionnaires were first developed in English and then translated into nine different languages (Urdu, Afrikaans, IsiXhosa, Romanian, Filipino (Tagalog), Sinhala, Tamil, Vietnamese, and Twi) guided by the WHO Guidelines on Translation. The translation included two independent forward translations into each language by professionals who met the established requirements, including being native speakers of the target language with excellent knowledge of English and experience with translating for research purposes. Then, an expert panel consisting of at least two experts in the field of maternal and child health and/or mental health, at least one EBLS co-investigator, and the two translators, suggested improvements in culturally adapted translation and addressed ambiguities. The protocol of this study provides further details of the data collection procedures [31].

### 2.2. Ethics

Ethical approval was obtained following national specific procedures in each of the eight participating study sites and the coordinating site prior to the start of data collection.

### 2.3. Sample

A convenience sampling method was employed to recruit participants through direct contact by the fieldworkers in the clinic waiting room, or by a health worker who would then refer potential participants to the fieldworkers. Recruitment strategies were adapted in each site. For instance, in South Africa, the EBLS team anticipated low participation rates because another study had recently recruited participants with similar profiles in the same area. To address this, the team implemented a door-to-door recruitment approach. In Romania, participants had to be accessed through local healthcare providers.

Pregnant women were invited to participate in the study if they met the following conditions: (i) were in their third trimester of pregnancy (i.e., weeks 29–40); (ii) were aged 18 and over; (iii) were residing within the study area and had no plans to migrate during the first three months post-birth; and (iv) were able to give informed consent. The total baseline sample consisted of 1208 participants (150 approx. per site) that were on average 28.27 years old (SD = 5.81 years, range: 18–48 years). For further participant demographic characteristics, see Murray et al. (2022) [30].

### 2.4. Procedure

Informed consent was obtained from all participants involved in the study. Following the guidelines by the WHO’s Research Ethics Committee [31], alternative methods of obtaining consent were provided as needed, including audio recordings for individuals unable to write and thumbprint verification on the consent form for those who are illiterate.

Female fieldworkers interviewed participants after receiving 40 h of standardized in-person training [31]. Recruitment and baseline data collection started in January 2019 and ended in December 2019. Interviews combined Computer-Aided Personal Interviews (CAPI) and Computer-Assisted Self-Interviewing (CASI) for the more sensitive items. The interview settings varied by site and included the project office or clinic designated space primarily and the participants’ houses. The settings for the interviews and the recruitment strategies were adapted to meet the specific needs of each site. For example, in Sri Lanka and the Philippines, severe weather occasionally made it difficult to arrange interviews, requiring flexibility in scheduling and location selection.

## 3. Measures

### 3.1. Neighborhood Characteristics Scales

To measure neighborhood characteristics, an instrument was specifically developed by the EBLS Consortium, with 18 items (See Supplementary Materials: Table S1) adopted from existing validated measures as described below.

Neighborhood cohesion: Five items from Mujahid et al.’s [10] scale asked about the presence of support and help, positive relationships, trust, and shared values within the neighborhood. Responses were measured on a four-point Likert scale ranging from 1 (strongly agree) to 4 (strongly disagree) (Cronbach’s alpha = 0.83).

Intergenerational Closure: Four items from Sampson and colleagues [18] assessed intergenerational closure, measured on a four-point Likert scale ranging from 1 (strongly agree) to 4 (strongly disagree). Items asked about the presence of adults in the neighborhood that watch out for children, if these adult figures can be looked up to by children, and the relationship among parents (Cronbach’s alpha = 0.63).

Neighborhood disorder: Four items from the Neighborhood Disorder Observation Scale [9] assessed the dimensions of neighborhood disorder, with a four-point response scale ranging from 1 (not a problem) to 4 (large problem). Items to measure neighborhood disorder asked about litter in the streets, smells and fumes, noise from traffic or other homes, and traffic and road safety (Cronbach’s alpha = 0.80).

Social disorder: Five items from the Neighborhood Disorder Observation Scale [9] assessed the dimensions of social disorder, with a four-point response scale ranging from 1 (not a problem) to 4 (large problem). Items inquired about vandalism, people being drunk on the streets, gangs, fights and arguments on the streets, and whether people are afraid of going out at night (Cronbach’s alpha = 0.87).

### 3.2. Nomological Net Measures

Perceived Stress Scale (PSS) [32]: This 10-item instrument measures how stressful certain life situations are rated by respondents during the last month. Responses were measured on a four-point Likert scale ranging from 1 (not at all) to 4 (nearly every day) (Cronbach’s alpha = 0.76).

WHO (Five) Well-Being Index (1998 version) [33]: The WHO Well-Being Index is a five-item screening questionnaire used to determine the subjective psychological well-being of respondents within the past two weeks. Response categories include a six-point Likert scale ranging from 1 (at no time) to 4 (all the time) (Cronbach’s alpha = 0.84).

Patient Health Questionnaire (PHQ-9) [34]: To measure the severity of depressive symptoms in the last two weeks, the PHQ-9 employs nine items covering anhedonia, dysphoria, sleep disturbances, fatigue, changes in eating, low self-esteem, concentration difficulties, hypo-or-hyperactive behaviors, and suicide ideation. Response categories include four points ranging from 1 (not at all) to 4 (nearly every day) (Cronbach’s alpha = 0.76).

### 3.3. Analytical Strategy

#### 3.3.1. Factorial Validity and Measurement Invariance

To evaluate the factorial structure of the selected measures, we conducted a confirmatory factor analysis including four factors: neighborhood cohesion, intergenerational closure, neighborhood disorder, and social disorder. This factorial structure was fitted individually for each country. Correlations were permitted between the factors, with non-significant correlations removed from the final models. Model identification was specified using the ‘marker indicator’ method, whereby the loading of one item was fixed to 1 and the variance of the factor was freely estimated. To account for the categorical nature of the items, all models were estimated using the weighted least squares with the robust means and variances (WLSMV) estimator. Model fit was evaluated using the comparative fit index (CFI), the Tucker–Lewis index (TLI), the root mean square error of approximation (RMSEA), and the root mean square residuals (SRMR). Good model fit was indicated by CFI ≥ 0.95, TLI ≥ 0.95, RMSEA ≤ 0.06, and SRMR ≤ 0.08; adequate model fit was indicated by CFI ≥ 0.90, TLI ≥ 0.90, RMSEA ≤ 0.08, and SRMR ≤ 0.08 [35,36,37].

To examine whether the measures operated equivalently across countries, we conducted measurement invariance tests. Importantly, on several of the items, category 4 of response was not endorsed by any participant or was endorsed by very few participants, in at least some of the countries. Therefore, to balance the aim of comparing responses across all eight countries with the aim to maximize scale integrity (i.e., retaining the entire spectrum of possible response choices), we employed the following rule: we collapsed categories 3 and 4 where no more than 10 people endorsed response category 4 in at least four of the countries (i.e., 50% of the sites) or where category 4 was not endorsed by any participant in at least one country (because such a case impedes invariance testing). Consequently, the social cohesion and intergenerational closure subscales contained three categories for the purpose of this analysis, whereas four categories were retained for neighborhood and social disorder.

Measurement invariance was tested at three levels: configural, metric, and scalar. First, the configural model—serving as the baseline—inspected the extent to which the same factorial structure (i.e., pattern of loading) was applicable across the eight sites, namely whether the item-to-factor relationships were observed across sites. This is the weakest form of invariance. Secondly, the metric model was specified as a nested model within the configural model, with loadings of corresponding items constrained to be equal across groups. Finally, the scalar model was specified as a nested model within the metric model, with each threshold of each item constrained to be equal across groups, allowing latent factor means to be compared. To ensure model identification, the configural and metric models contained several parameters that were fixed in all groups: (a) the means of all factors were fixed to zero and (b) the item scale factors were fixed to 1. The scalar model retained these fixed parameters only for the ‘reference’ group. As there was no specific rationale for choosing one country over another as the ‘reference’ group, this was given by the first country in an alphabetically ordered list of country names (i.e., Ghana).

Configural invariance was achieved if the configural model fitted the data well according to the CFI, TLI, RMSEA and SRMR. Metric invariance was achieved if the fit indicators did not deteriorate by values greater than the following: 0.10 for CFI and TLI, 0.015 for RMSEA, and 0.03 for SRMR [38]. Scalar invariance was achieved if the fit indictors did not deteriorate by values greater than the following: 0.10 for CFI and TLI, 0.015 for RMSEA, and 0.01 for SRMR [38]. In the case of non-invariance, partial invariance was sought by releasing constraints on the loadings (in the metric model) or thresholds (in the scalar model), under guidance from the model modification indices. To retain the maximum level of invariance possible, such modifications were only performed for the countries where non-invariance was observed (i.e., it was possible for an item’s thresholds to exhibit invariance across six out of eight countries).

All models were conducted using the software Mplus v.8.8 [39]. To aid the identification of the best possible solution, we employed 10 random starts and 10,000 iterations.

#### 3.3.2. Nomological Networks

We obtained Pearson’s correlations between all the variables for each of the study sites. Then, we used qgraph with R version 4.3.1. [40] to create diagrams with the aim of illustrating the association between the four neighborhood characteristics that concern the current study and the maternal outcomes of interest and compare these associations across countries. In the figures, the edge thicknesses are proportional to the magnitude of the Pearson’s correlations between each variable, which allows a visual comparison across study sites.

## 4. Results

### 4.1. Descriptive Statistics

Table 1 and Table 2 provide descriptive statistics for each item of the neighborhood scales and maternal well-being outcomes, respectively, by country, showing some variation in mean levels across sites.

### 4.2. Internal Consistency

To investigate whether the questionnaire items were amenable to grouping into themes (i.e., the proposed conceptual factors), we conducted internal consistency tests. Cronbach’s alpha reliability values were acceptable in all countries (See Supplementary Materials: Table S2). When examining internal consistency for each of the four dimensions for each country, we noted that Ghana had low internal reliability for intergenerational closure.

### 4.3. Factorial Validity

#### Factorial Structure

To evaluate whether the questionnaire items reflected underlying dimensions of neighborhood cohesion, intergenerational closure, neighborhood disorder, and social disorder, we conducted a four-factor confirmatory factor analysis. The four-factor structure showed an overall good fit to the data (Table 3). Fit indicators suggested excellent fit to data from six countries: Jamaica, Philippines, Romania, South Africa, Sri Lanka, and Vietnam. However, for Pakistan, the SRMR value fell below its cut-off for adequate fit, with all other fit indicators suggesting excellent fit, and for Ghana, the model fit indices fell slightly below the thresholds for excellent fit, but indicated adequate fit. To avoid model fit improvements driven by sample peculiarities, we accepted this model without further modifications.

No cross-loadings were observed based on inspection of the model modification indices, except for item 3 (i.e., “People in my neighborhood generally get along well with each other.”) in Ghana, which showed a potential cross-loading from its designated factor of poor neighborhood cohesion to the factor of neighborhood disorder. Given that the model showed overall good fit to the data and to avoid data-driven results, we did not modify this model further.

The pattern of inter-factor correlations indicates country-specific patterns (See Supplementary Materials: Table S3). Strong positive correlations were noted across all eight sites between the neighborhood and the social disorder scales, and between the scales referring to poor neighborhood cohesion and low intergenerational closure. In spite of the high correlations, the four scales are conceptually dissimilar and, therefore, amalgamating items of correlated scales into single over-arching total scores (e.g., ‘overall disorder’ and ‘social capital’) risks a loss of sensitivity with regard to potential cultural differences across countries in terms of the presence of the four neighborhood characteristics and their potential impact on pregnant women’s well-being. Retaining four factors was further supported by technical indicators. Specifically, a lower good model fit was produced by alternative models comprising (a) two single-order factors applied to items pooled across inter-related scales or (b) a higher structure with second-order factors for disorder (fitted to the first-order factors of neighborhood and social disorder) and social closeness (fitted to the first-order factors of social cohesion and intergenerational closure). Hence, the original four factor structure was retained.

### 4.4. Cross-Country Invariance

The factorial structure was similar across all eight countries, enabling measurement invariance tests spanning all locations (See Supplementary Materials: Table S4). Therefore, we proceeded with invariance tests at three levels: configural (i.e., same factorial structure across sites), metric (i.e., same factor loadings for corresponding items across sites), and scalar (i.e., same threshold levels for corresponding items across sites). The configural model exhibited a good fit with the data, indicating that configural invariance was achieved. The addition of constraints imposed on factor loadings did not produce deteriorations in model fit values greater than the cut-off values proposed by Chen [38]. Hence, full metric invariance was achieved, showing that the relative importance of all items in relation to their corresponding factors was retained across all eight countries (See Supplementary Materials: Table S5). Regarding neighborhood cohesion, stronger loadings were observed for the items about social harmony (i.e., help, support, and ‘getting along’) than for the items about mutual trust and shared values. For intergenerational closure, higher loadings were observed for the items about residents’ reliability than for the items about how well parents know other adults or children in the neighborhood. Regarding neighborhood disorder, higher loadings were exhibited by the items about the presence of litter and smell and fumes, whereas slightly lower loadings were exhibited by the items about road safety and noise from traffic and homes. Finally, for social disorder, the highest loadings were in relation to the items about vandalism and the presence of gangs, with slightly lower loadings in relation to items reflecting street safety.

The addition of constraints, imposed onto item thresholds, led to a significant deterioration in model fit. Hence, under guidance from model modification indices, threshold constraints were removed in iterative steps. In the final model, threshold constraints were removed for item 5 in the sample from Ghana, for items 14, 15, and 17 (all related to social disorder) in the sample from Jamaica, for item 5 (neighborhood cohesion) and item 6 (intergenerational closure) in the sample from Pakistan, and for item 14 (social disorder) in the samples from Philippines and South Africa—see Table 1 for item numbers. Under this partial scalar model, all the thresholds of the remaining 13 items were invariant across all eight groups (See Supplementary Materials: Table S6).

Significant variation in people’s perceptions of cohesion, intergenerational closure, and disorder was observed in all countries (See Supplementary Materials: Table S5). Relative to levels observed in the reference group (i.e., Ghana), neighborhood cohesion was significantly poorer in Jamaica and stronger in Sri Lanka and Vietnam, intergenerational closure levels were significantly lower in Romania, Sri Lanka, and Vietnam, neighborhood disorder levels were significantly higher in Jamaica, the Philippines, South Africa, and Sri Lanka, and social disorder levels were significantly higher in Jamaica and South Africa and lower in Pakistan and Romania.

### 4.5. Nomological Network

**Figure 1:**
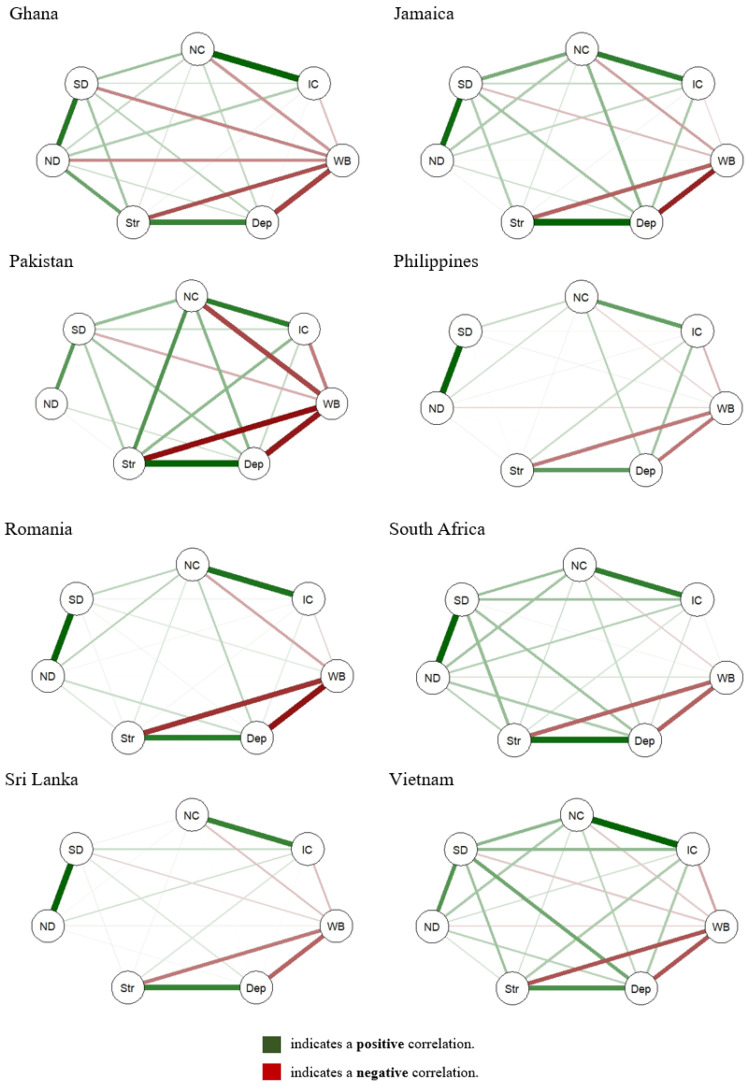
Nomological nets for neighborhood characteristics and maternal outcomes for each of the eight countries. Abbreviations. NC: neighborhood cohesion; IC: intergenerational closure; WB: well-being; Dep: depression; Str: stress; ND: neighborhood disorder; SD: social disorder. Note 1. Edge thicknesses are proportional to the magnitude of the Pearson’s correlations between variables (i.e., ticker edge means stronger correlation, and vice versa).

Having established that the measures functioned equivalently across sites to a considerable extent, we investigated correlation patterns between neighborhood characteristics and maternal well-being. The nomological networks illustrate the correlations between the four neighborhood factors—i.e., neighborhood cohesion, intergenerational closure, neighborhood disorder, and social disorder—and the three maternal outcomes (depressive symptoms, stress, and well-being). See Supplementary Materials: Table S7. The variation in correlation strengths and directions across countries suggests that the relationships between neighborhood factors and maternal health outcomes are context-dependent (Figure 1).

Across all sites, positive correlations are observed between neighborhood cohesion and well-being, indicating that higher neighborhood cohesion is consistently associated with better maternal well-being. This relationship appears to be particularly strong in Vietnam and South Africa. Conversely, neighborhood disorder and social disorder show negative correlations with well-being across most sites, reinforcing the idea that higher levels of neighborhood and social disorder are linked to poorer maternal well-being. This pattern is most pronounced in Pakistan and Romania.

The associations between neighborhood factors and depressive symptoms also exhibit variability. In Pakistan, Romania, and Ghana, neighborhood disorder and social disorder have strong positive correlations with depressive symptoms. This pattern is less pronounced in Sri Lanka and the Philippines. Similarly, stress exhibits notable positive correlations with neighborhood disorder and social disorder in multiple sites, with the strongest associations appearing in Pakistan and Romania. In South Africa and Vietnam, these correlations are weaker.

Intergenerational closure appears to have weaker and more variable associations across countries. While some positive correlations with well-being are noted, they are not as strong as those observed for neighborhood cohesion.

## 5. Discussion

Using data from the EBLS study, we assessed the conceptual and measurement equivalence of the community domains of neighborhood cohesion, intergenerational closure, and neighborhood and social disorder, testing for measurement invariance across eight LMICs. Next, we examined patterns of associations with prenatal maternal stress, well-being, and depressive symptoms. Our findings confirm that the studied scales reliably measure neighborhood cohesion, intergenerational closure, and neighborhood and social disorder across eight diverse LMICs. Additionally, our results underscore the important role of neighborhood characteristics in maternal well-being during pregnancy, with neighborhood cohesion serving as a protective factor against stress and depression, while disorder increases risk. However, the strength of these associations varies by country, highlighting the influence of sociocultural and environmental factors on maternal health outcomes.

Our findings from the invariance analysis suggest that the measures selected to capture neighborhood characteristics are relatively consistent across the eight different countries. This means that the conceptual and measurement equivalence of the community domains of neighborhood cohesion, intergenerational closure, and neighborhood and social disorder is comparable across eight diverse LMICs. Therefore, the use of the scale in question to measure these neighborhood characteristics in LMICs can be supported. However, it should also be noted that the data from the Ghana and Pakistan sites did not fit the data excellently across all fit indicators. The findings from our factorial structure analysis reveal an excellent fit to the data for the remaining countries (i.e., Jamaica, Philippines, Romania, South Africa, Sri Lanka, and Vietnam). The variation in model fit across countries, particularly the lower fit observed in Ghana and Pakistan, may be linked to multiple factors. One possibility is that some items were less culturally relevant in specific contexts. For example, in Pakistan, alcohol consumption is highly restricted, which may have influenced responses to the corresponding item (“There are people being drunk on the streets”). Additionally, respondents’ familiarity with their neighborhood in Pakistan may vary, potentially affecting responses to items assessing neighborhood perceptions. Despite following a rigorous translation protocol, subtle differences in interpretation across languages cannot be entirely ruled out. Previous studies have found that other constructs, such as perceived stress [41] and prenatal attachment [29], demonstrated good model fit in Pakistan but lower fit in Ghana, suggesting that cultural and contextual factors may influence how certain constructs are understood and measured across different settings.

Furthermore, we noted that the last response category of some items was not endorsed at some of the sites. This could indicate that some sites were more successful in recruiting pregnant women from a wider range of neighborhoods than other sites, or that there are genuine sociocultural differences in how neighborhoods are perceived by pregnant women. Further studies on this topic could help to elucidate this point and consolidate the interpretability and deployment of these promising scales. The neighborhood-related scores were driven by items about social harmony and residents’ reliability to a greater extent than items about shared values, mutual trust, and resident familiarity. This was also noted in relation to the dimensions of disorder, where vandalism, gang presence, and litter were more important than noise from traffic and homes or street safety. This suggests that, across all countries, feeling that one has ‘good neighbors’ created an ineffable social support net that transcended potential perceptions of personal value disparities or the general inconveniences of living in close proximity to others.

From a methodological perspective, our study highlights the challenges associated with measuring neighborhood-level variables across diverse settings. Consistent with prior concerns [13,25,26], the lack of validated instruments for assessing neighborhood characteristics across cultural contexts presents a limitation for cross-country comparisons. However, our findings emphasize the need for establishing measurement invariance to facilitate meaningful cross-context assessments [27,28].

Our findings underscore the relevance of neighborhood characteristics in shaping maternal well-being during pregnancy. These results align with Bronfenbrenner’s bioecological framework [7,8], which posits that human development is embedded within nested social structures. In this study, we observed that neighborhood-level factors, including social cohesion, disorder, and intergenerational closure, significantly influenced maternal stress and well-being, supporting the premise that these constructs operate within broader ecological systems.

To illustrate the associations between neighborhood characteristics and maternal well-being, we constructed nomological networks across eight culturally diverse sites.

The study contributes to the growing body of literature suggesting that neighborhood characteristics do not function in isolation but rather interact with individual-level factors [11]. Our findings highlight that the protective role of neighborhood cohesion was associated with lower maternal stress levels, in line with past research on community participation and maternal mental health [13,17]. Additionally, all sites exhibited positive correlations between neighborhood cohesion and well-being. However, this relationship appears to be particularly strong in Vietnam and South Africa, suggesting that in these settings, a strong sense of community and support networks may have a more pronounced protective effect on maternal mental health. The negative associations between neighborhood disorder and social disorder with well-being were more pronounced in Pakistan and Romania, indicating potential stronger detrimental effects in these contexts. This may reflect heightened social stressors or safety concerns, which could exacerbate maternal distress.

Similarly, we found that higher levels of neighborhood and social disorder were linked to increased adverse maternal outcomes, corroborating emerging evidence on the detrimental effects of disorder during the perinatal period [9,21,22]. However, the associations between neighborhood factors and depressive symptoms varied across sites. Stronger correlations between neighborhood and social disorder and depressive symptoms in Pakistan, Romania, and Ghana suggest that maternal depression in these countries is particularly sensitive to perceptions of neighborhood safety and social cohesion. This pattern is less pronounced in Sri Lanka and the Philippines, possibly indicating that other resilience factors mitigate the effects of neighborhood adversity in these regions. However, a previous study on the validation and cross-country invariance of Patient Health Questionnaire-9 (PHQ-9) using EBLS data [30] should be considered in the interpretation of these results. The study in question found that while the PHQ-9 yields reliable scores in antenatal populations across the same settings as in this study, the interpretation of these scores may vary depending on the cultural context. This highlights the need to consider local understandings of depressive symptoms to ensure that the measure accurately captures the unique ways antenatal depression is experienced and expressed in each setting. Similarly, while stress exhibited stronger associations with neighborhood disorder and social disorder in Romania and Pakistan, in South Africa and Vietnam, these correlations were weaker, potentially implying that maternal stress may be influenced by other environmental or sociocultural factors beyond neighborhood conditions.

Intergenerational closure, a relatively underexplored construct in maternal health research, also emerged as a significant factor influencing maternal well-being. This finding expands existing ecological theories by suggesting that dense social networks between adults and children in a neighborhood may contribute to emotional and psychological resilience among pregnant women [18]. Intergenerational closure showed weaker and more variable associations with maternal outcomes across sites. Cultural differences in child-rearing practices, varying definitions of community responsibility, and different levels of urbanization could all influence how intergenerational closure manifests and affects maternal well-being. Additionally, the effectiveness of intergenerational closure might depend on the quality of these adult–child relationships rather than just their presence. For example, in Vietnamese urban high-income families, “tiger parenting” practices are common, which significantly impacts intergenerational closure in neighborhoods [42,43]. Parents in these communities often prioritize their own children’s academic performance over community involvement, leading to a reduced likelihood of adults watching out for other children in the neighborhood. This stems from an assumption that each parent has their own specific plan for their child, and the responsibility of “watching out for children” is typically viewed as a family duty rather than a community one.

While intergenerational closure may contribute to maternal well-being, its impact appears less consistent than broader neighborhood cohesion, indicating that its role may depend on additional contextual factors. The relationship between intergenerational closure and maternal outcomes might be mediated by factors such as cultural norms, socioeconomic conditions, and existing social support systems. As with any self-report measure, responses to the intergenerational closure scale could be subject to social desirability bias. For instance, in the Vietnamese context, cultural emphasis on harmony and respect is important [44]. This might result in respondents overreporting positive aspects of their neighborhood or being hesitant to express negative perceptions. Future research should further explore this dimension to understand its mechanisms and potential implications for intervention strategies.

Overall, while certain general patterns emerged in our study—such as the protective effect of neighborhood cohesion and the detrimental impact of neighborhood and social disorder—these associations are not uniform across countries. Sociocultural differences, variations in urban infrastructure, and differing levels of social support likely shape these relationships.

Furthermore, it should be noted that nomological networks do not fully control for confounding factors, which may influence the findings and limit the ability to establish meaningful causal relationships. For instance, variables such as the number of children could impact maternal mental health [45], potentially confounding the observed associations. Without accounting for these factors, the results may partially reflect the influence of unmeasured variables rather than direct relationships between the studied constructs. Future research should incorporate these additional variables to provide a more comprehensive and precise understanding of maternal well-being. Additionally, the differences in mental health assessment timeframes across the measures used in this study may introduce potential biases that should be considered when interpreting the findings. While the Perceived Stress Scale (PSS) assesses stress levels over the past month, the WHO Well-Being Index and Patient Health Questionnaire-9 (PHQ-9) focus on the past two weeks. These variations in reference periods may lead to discrepancies in how respondents recall and report their mental health experiences, potentially influencing observed associations between stress, well-being, and depressive symptoms. Future research should account for these differences. It is important to consider that other characteristics could have influenced the results. For instance, the choice of interview settings can significantly influence data collection, particularly when addressing sensitive topics. Additionally, sensitive issues, which have the potential to cause distress to participants or researchers [46], could lead to interviewer bias. These factors should be taken into account when interpreting the findings of this study, since the interview setting was adapted to each of the eight participating sites.

To ensure linguistic and conceptual equivalence in assessing neighborhood characteristics across diverse cultural contexts, we followed the WHO Guidelines on Translation. This process involved two independent forward translations into each target language by qualified professionals, followed by a review from an expert panel consisting of specialists in maternal and child health and/or mental health, at least one EBLS co-investigator, and the translators. This rigorous approach helped to resolve ambiguities, refine cultural adaptations, and enhance the validity of the survey items. Despite these efforts, some cultural biases in interpreting neighborhood characteristics may persist, highlighting the importance of future research exploring how neighborhood perceptions vary across different sociocultural settings.

It should be noted that the definition of “neighborhood” can vary across cultures [47]. These variations may influence how participants perceive and report their experiences, potentially leading to biases in the study’s findings. Additionally, differences in how mental health is assessed over time—shaped by cultural norms regarding disclosure and the timeframe for evaluating well-being—can affect the consistency and accuracy of mental health reports [48,49]. Recognizing these cultural variations in both neighborhood definitions and mental health assessment timeframes is important to understand the potential biases and limitations of the study.

Testing psychometric properties of instruments used in the context of maternal well-being across different cultures is crucial for improving global evidence on critical aspects that affect pregnant women’s health and well-being. Given that women are particularly vulnerable during pregnancy and the perinatal period [50], this is a unique window of opportunity for policies to have a large positive impact on changing maternal trajectories. Furthermore, since neighborhood-level factors have the potential to impact women’s health [1,2,3], pregnancies and perinatal health outcomes, this period is key for policies that aim to positively impact children’s development from the earliest stages. Our findings suggest that improving neighborhood cohesion and reducing disorder, as well as promoting intergenerational closure, could serve as key intervention points for policymakers. In the context of LMICs, specific policy recommendations should focus on enhancing community-based health programs that provide maternal support services, increasing investments in safe public spaces for social interaction, and fostering partnerships between local governments and non-governmental organizations to improve neighborhood conditions. Strengthening local governance, investing in community resources, and developing culturally adaptable intervention frameworks could contribute to improved maternal and child health outcomes worldwide.

Further work that tests whether the observed differences are significant and whether they are due to cultural disparities will greatly advance the understanding of the role played by neighborhood characteristics in maternal well-being in different countries.

### Strengths and Limitations

This study has several strengths. First, the findings contribute to an increase in the amount of evidence on sociometric assessments of instruments measuring neighborhood characteristics. Second, results from the measurement invariance suggest that the instruments used to measure neighborhood characteristics are adequate for use in LMICs in diverse world regions. Third, the nomological networks increase knowledge on how social and material features of neighborhoods are associated with constructs of maternal well-being outcomes across diverse societies.

However, some limitations should also be acknowledged. First, while the study examines perceived neighborhood characteristics, it was not possible to link the subjective perceptions of participants to neighborhood-level characteristics obtained from, for example, census data. Second, it was necessary to adapt the sampling strategies in each of the study sites for both cultural and practical reasons. However, we tailored recruitment approaches to align with local cultural norms, community dynamics, and the specific environmental and situational factors to increase participant engagement and ensure the reliability of data collected. Third, our sampling procedures were non-probabilistic, which may limit the generalizability of our findings to broader populations. Fourth, despite the utility of nomological networks to assess associations between relevant variables, this is not an in-depth analysis that controlled for confounding variables to establish meaningful associations. Fifth, it should be noted that the three instruments that captured adverse mental health outcomes (i.e., stress, depression, and well-being) considered slightly different timeframes (i.e., last month, last 2 weeks), which might have led to omitting relevant information that occurred at a different moment of pregnancy. Sixth, our study did not account for other important neighborhood characteristics, such as access to green spaces, community resources, overcrowding, and healthcare access. Seventh, the relatively small sample size of approximately 150 participants per country may constrain the statistical power for cross-country comparisons and structural equation modeling. However, previous EBLS studies have demonstrated the feasibility of using similar sample sizes, supported by Monte Carlo power analyses (see [30]). While our findings remain robust within these constraints, future research with larger and more diverse samples would further strengthen the generalizability of the results. Finally, the definition of “neighborhood” may vary across participants and across cultures.

## 6. Conclusions

Our findings suggest that the studied scales measured the domains of neighborhood cohesion, intergenerational closure, and neighborhood and social disorder comparably across the eight different sites and, therefore, present a valid approach to measure social and material features of neighborhoods in LMICs in diverse world regions. Moreover, our findings highlight the critical role of neighborhood characteristics in shaping maternal well-being during pregnancy across diverse LMICs. The results reinforce the importance of neighborhood cohesion as a protective factor against maternal stress and depression, while neighborhood and social disorder pose significant risks. However, the magnitude of these associations varies by country, suggesting that sociocultural and environmental factors influence how neighborhood conditions impact maternal health.

From a policy and intervention perspective, our study underscores the need to improve neighborhood cohesion and reduce disorder to enhance maternal health outcomes. Community-based programs, investments in safe public spaces, and stronger local governance could be instrumental in fostering supportive environments for pregnant women. Additionally, while our measurement invariance analysis supports the cross-cultural validity of the scales used, further research should refine these instruments to better capture neighborhood experiences in different cultural contexts. Future studies should also explore additional neighborhood factors, such as access to healthcare and community resources, to provide a more comprehensive understanding of their impact on maternal well-being. By addressing these gaps, research can contribute to evidence-based policies that promote maternal health and early childhood development on a global scale.

## Figures and Tables

**Table 1:** Neighborhood item and descriptive statistics by country.

	Ghana		Jamaica		Pakistan		Philippines		Romania		South Africa		Sri Lanka		Vietnam	
	M	(SD)	M	(SD)	M	(SD)	M	(SD)	M	(SD)	M	(SD)	M	(SD)	M	(SD)
Support	2.12	(1.09)	2.67	(1.10)	1.99	(1.13)	1.93	(0.85)	1.93	(0.53)	2.19	(1.08)	1.57	(0.63)	1.59	(0.69)
2. Help	2.12	(1.05)	2.56	(1.02)	2.00	(1.12)	1.80	(0.88)	1.79	(0.59)	1.97	(1.03)	1.66	(0.67)	1.57	(0.68)
3. Get along	1.74	(0.89)	2.81	(1.09)	1.58	(0.81)	1.80	(0.85)	1.75	(0.57)	2.11	(1.06)	1.67	(0.64)	1.63	(0.68)
4. Trust	2.63	(1.14)	3.08	(0.99)	1.97	(1.01)	2.15	(0.97)	1.87	(0.55)	2.51	(1.12)	2.16	(0.84)	1.88	(0.81)
5. Values	1.59	(0.90)	3.04	(0.97)	2.92	(1.15)	2.10	(0.94)	2.17	(0.63)	2.71	(1.11)	2.17	(0.81)	2.14	(0.75)
6. Look up to	1.73	(0.99)	1.79	(0.91)	2.72	(1.21)	1.80	(0.89)	1.75	(0.58)	1.93	(1.07)	1.79	(0.65)	1.84	(0.75)
7. Adults watch out	1.89	(1.07)	1.92	(0.97)	2.74	(1.27)	1.52	(0.71)	1.93	(0.70)	1.70	(0.94)	2.08	(0.89)	2.31	(1.04)
8. Parents know friends	1.96	(0.98)	2.14	(1.0)	1.90	(0.98)	1.51	(0.80)	1.82	(0.63)	1.65	(0.93)	1.95	(0.71)	2.09	(0.95)
9. Parents know each other	1.77	(0.91)	1.56	(0.78)	1.54	(0.79)	1.43	(0.67)	1.78	(0.67)	1.47	(0.80)	1.74	(0.61)	1.53	(0.69)
10. Litter	1.81	(1.08)	2.27	(1.17)	2.26	(1.32)	2.60	(1.17)	1.82	(0.98)	2.48	(1.23)	2.50	(1.26)	1.75	(0.82)
11. Smells and fumes	1.73	(1.05)	2.16	(1.21)	2.14	(1.32)	2.47	(1.25)	1.81	(1.03)	2.28	(1.25)	2.26	(1.29)	1.48	(0.79)
12. Noise	2.10	(1.13)	2.03	(1.11)	1.75	(1.13)	2.17	(1.17)	1.78	(0.97)	2.23	(1.20)	1.93	(1.07)	1.71	(0.92)
13. Poor safety	1.93	(1.11)	1.98	(1.08)	1.99	(1.18)	2.13	(1.24)	2.05	(1.04)	2.25	(1.16)	2.10	(1.17)	1.45	(0.78)
14. Vandalism	1.84	(1.11)	1.66	(0.97)	1.39	(0.89)	2.30	(1.26)	1.38	(0.82)	2.16	(1.23)	1.95	(1.19)	1.41	(0.70)
15. Drunk people	1.97	(1.11)	1.76	(0.97)	1.43	(0.87)	2.40	(1.23)	1.65	(0.93)	2.75	(1.23)	2.24	(1.22)	1.53	(0.75)
16. Gangs	1.86	(1.10)	2.45	(1.25)	1.32	(0.82)	1.91	(1.25)	1.45	(0.82)	2.99	(1.29)	2.08	(1.28)	1.41	(0.73)
17. Arguments	1.95	(1.12)	2.66	(1.14)	1.54	(0.94)	2.10	(1.19)	1.30	(0.73)	2.83	(1.23)	2.01	(1.16)	1.57	(0.78)
18. Afraid of going out	2.26	(1.29)	2.20	(1.28)	1.86	(1.12)	1.96	(1.17)	1.29	(0.70)	2.93	(1.28)	1.76	(1.16)	1.33	(0.70)

Abbreviations. M: mean; SD: standard deviation.

**Table 2:** Maternal health items and descriptive statistics by country.

	Ghana		Jamaica		Pakistan		Philippines		Romania		South Africa		Sri Lanka		Vietnam	
	M	(SD)	M	(SD)	M	(SD)	M	(SD)	M	(SD)	M	(SD)	M	(SD)	M	(SD)
Perceived stress																
Upset	1.66	(0.84)	2.24	(1.01)	1.74	(1.00)	1.83	(0.90)	1.44	(0.50)	1.99	(1.07)	1.70	(0.62)	1.43	(0.52)
2. Control	1.72	(0.94)	2.04	(1.14)	1.55	(0.96)	1.59	(0.78)	1.39	(0.54)	1.69	(0.94)	1.37	(0.66)	1.41	(0.53)
3. Nervous	1.91	(0.93)	2.16	(1.10)	1.89	(1.17)	2.06	(0.98)	1.89	(0.60)	1.93	(1.11)	1.62	(0.64)	1.67	(0.65)
4. Confident	2.75	(0.86)	2.38	(1.04)	2.16	(1.16)	1.97	(1.12)	1.72	(0.86)	2.32	(1.25)	1.94	(1.20)	2.29	(1.10)
5. Things my way	2.86	(0.97)	2.70	(1.04)	2.47	(1.16)	2.45	(1.08)	1.69	(0.80)	2.43	(1.14)	2.42	(1.16)	2.35	(1.04)
6. Coping	1.81	(0.83)	2.30	(1.10)	1.82	(1.05)	1.63	(0.87)	1.95	(0.79)	2.11	(1.11)	1.66	(0.88)	1.60	(0.77)
7. Control	2.88	(0.92)	2.58	(1.01)	2.46	(1.11)	2.52	(1.10)	1.78	(0.92)	2.52	(1.20)	2.35	(1.19)	2.01	(1.07)
8. On top of things	2.96	(1.00)	2.77	(1.07)	3.73	(0.70)	2.45	(1.17)	1.59	(0.75)	2.37	(1.16)	2.65	(1.24)	1.79	(0.95)
9. Angered	1.81	(0.91)	2.46	(1.06)	1.75	(0.90)	2.01	(1.06)	1.74	(0.66)	2.42	(1.19)	1.66	(0.80)	1.65	(0.60)
10. Difficulties	1.63	(0.90)	2.13	(1.14)	1.86	(1.15)	1.83	(1.04)	1.27	(0.49)	1.91	(1.14)	1.64	(0.91)	1.33	(0.59)
Well-being																
Cheerful	2.56	(1.69)	3.28	(1.46)	2.66	(1.85)	3.34	(1.66)	3.86	(0.79)	3.04	(1.57)	3.50	(1.52)	3.43	(1.44)
2. Calm	2.84	(1.70)	2.83	(1.56)	2.76	(1.88)	3.16	(1.69)	3.62	(1.00)	3.55	(1.52)	3.21	(1.55)	3.44	(1.49)
3. Active	2.47	(1.63)	2.78	(1.57)	1.99	(1.94)	3.36	(1.72)	3.31	(1.17)	3.40	(1.55)	3.03	(1.63)	2.89	(1.57)
4. Fresh	2.39	(1.65)	2.56	(1.67)	2.63	(2.04)	3.25	(1.74)	2.91	(1.35)	3.23	(1.64)	2.99	(1.69)	2.92	(1.62)
5. Filled	2.05	(1.59)	2.63	(1.60)	2.34	(2.04)	3.03	(1.80)	3.90	(0.98)	3.11	(1.72)	3.26	(1.61)	3.51	(1.52)
Depressive symptoms																
Pleasure	0.89	(0.94)	1.32	(1.07)	1.10	(1.19)	1.07	(0.97)	0.65	(0.59)	1.19	(1.14)	0.70	(0.75)	0.65	(0.61)
2. Hopeless	0.77	(0.96)	1.03	(1.04)	0.91	(1.18)	0.80	(0.95)	0.27	(0.52)	0.63	(1.00)	0.63	(0.80)	0.33	(0.58)
3. Sleep	1.38	(0.99)	1.43	(1.15)	1.35	(1.36)	1.39	(0.99)	1.18	(0.88)	1.37	(1.17)	1.04	(0.88)	1.20	(0.85)
4. Energy	1.39	(0.89)	1.67	(1.05)	2.05	(1.16)	1.22	(0.93)	1.18	(0.76)	1.08	(1.13)	1.04	(0.87)	0.99	(0.77)
5. Eating	1.01	(0.99)	1.26	(1.14)	1.39	(1.34)	0.92	(1.07)	0.85	(0.82)	1.12	(1.16)	0.72	(0.83)	0.81	(0.87)
6. Failure	0.65	(0.91)	0.55	(0.97)	0.37	(0.84)	0.66	(0.92)	0.19	(0.49)	0.33	(0.79)	0.30	(0.58)	0.27	(0.55)
7. Concentration	0.52	(0.85)	0.71	(1.10)	0.37	(0.86)	0.66	(0.93)	0.41	(0.75)	0.38	(0.86)	0.30	(0.64)	0.51	(0.70)
8. Moving	0.48	(0.75)	0.73	(0.99)	0.59	(0.93)	0.56	(0.86)	0.35	(0.58)	0.47	(0.91)	0.47	(0.73)	0.40	(0.61)
9. Thoughts	0.32	(0.80)	0.36	(0.83)	0.13	(0.46)	0.17	(0.51)	0.03	(0.16)	0.25	(0.78)	0.17	(0.56)	0.06	(0.33)

Abbreviations. M: mean; SD: standard deviation.

**Table 3:** Model fit information for the four factor models fitted separately to each country.

	CFI	TLI	SRMR	RMSEA	RMSEA 90% CI
Ghana	0.94	0.93	0.11	0.08	[0.07, 0.10]
Jamaica	0.96	0.95	0.07	0.05	[0.03, 0.06]
Pakistan	0.98	0.98	0.10	0.07	[0.05, 0.08]
Philippines	0.99	0.98	0.07	0.05	[0.04, 0.07]
Romania	0.98	0.98	0.08	0.06	[0.04, 0.07]
South Africa	0.98	0.97	0.07	0.06	[0.04, 0.07]
Sri Lanka	0.99	0.99	0.07	0.05	[0.02, 0.06]
Vietnam	0.96	0.96	0.08	0.07	[0.05, 0.08]

Abbreviations. CFI: Comparative Fit Index; TLI: Tucker–Lewis Index; SRMR: Standardized Root Mean Square Residual; RMSEA: Root Mean Square Error of Approximation; CI: confidence interval.

## Data Availability

The raw data supporting the conclusions of this article will be made available by the authors on request.

## Supplementary Materials

The following supporting information can be downloaded at https://www.mdpi.com/article/10.3390/ijerph22030456/s1: Table S1: Instrument items; Table S2: Reliability by dimension and country; Table S3: Correlations between factors; Table S4: Model fit information for the configural, metric and scalar models; Table S5: Model parameters of the partial scalar model; Table S6: Factor means and variances as depicted by the partial scalar model; Table S7: Correlation matrix for nomological networks in the 8 countries.
