# Supplementary material for: Assessing Neighborhood Characteristics and Their Association with Prenatal Maternal Stress, Depressive Symptoms, and Well-Being in Eight Culturally Diverse Cities: A Cross-Sectional Study
Source: Int J Environ Res Public Health. 2025 Mar 20;22(3):456. doi: 10.3390/ijerph22030456 (PMC11942257; doi:10.3390/ijerph22030456)
Supplement: Supplementary file 1 [file ijerph-22-00456-s001.zip › ijerph-3485294-supplementary.pdf]

Table S1. Instrument items

	Strongly agree	Somewhat agree	Somewhat disagree	Strongly disagree
1 This is a neighbourhood where we all support each other	❍	❍	❍	❍
2 People around here are willing to help their neighbours.	❍	❍	❍	❍
3 People in my neighbourhood generally get along well with each other.	❍	❍	❍	❍
4 People in my neighbourhood can be trusted.	❍	❍	❍	❍
5 People in this neighbourhood share the same values.	❍	❍	❍	❍
6 There are adults in this neighbourhood that children can look up to.	❍	❍	❍	❍
7 You can count on the adults in this neighbourhood to watch out that children are safe and don't get in trouble.	❍	❍	❍	❍
8 Parents in this neighbourhood know their children's friends	❍	❍	❍	❍
9 Parents in this neighbourhood generally know each other.	❍	❍	❍	❍

	Not a problem	A small problem	Somewhat of a problem	A large problem
10 There is a lot of litter in the streets	❍	❍	❍	❍
11 There are smells and fumes from garbage, traffic and industry	❍	❍	❍	❍
12 There is a lot of noise from traffic or other homes	❍	❍	❍	❍
13 Traffic and road safety is poor	❍	❍	❍	❍
14 There is a lot of vandalism	❍	❍	❍	❍
15 There are people being drunk on the streets	❍	❍	❍	❍
16 There are gangs	❍	❍	❍	❍
17 There are fights and arguments on the streets	❍	❍	❍	❍
18 People are afraid of going out at night	❍	❍	❍	❍

Table S2. Reliability by dimension and country

	Cohesion	Closure	N. Disorder	S. Disorder
Ghana	.725	.371	.751	.811
Jamaica	.786	.536	.769	.753
Pakistan	.848	.640	.665	.669
Philippines	.819	.633	.878	.879
Romania	.830	.790	.768	.923
South Africa	.827	.659	.820	.861
Sri Lanka	.806	.666	.882	.930
Vietnam	.831	.729	.702	.773

Table S3. Correlations between factors

	Ghana	Jamaica	Pakistan	Philippines	Romania	South Africa	Sri Lanka	Vietnam
Poor neighborhood cohesion &								
Low intergenerational closure	.88***	.77***	.68***	.64***	.74***	.74***	.90***	.90***
Poor neighborhood disorder	*ns*	.27**	*ns*	.22*	.25**	.30***	*ns*	.29**
Social disorder	.29**	.41***	.30**	.24**	.31***	.33***	*ns*	.39***
Low intergenerational closure &								
Poor neighborhood disorder	*ns*	.26*	*ns*	*ns*	*ns*	.27**	.28***	*ns*
Social disorder	*ns*	.24*	.23*	*ns*	*ns*	.36***	.30***	.42***
Poor neighborhood disorder &								
Social disorder	.73***	.73***	.65***	.91***	.85***	.84***	.93***	.70**

Note. All coefficients are standardised by the variance of the latent variables. *** $p < .001$; ** $p < .01$; * $p < .05$; "ns" represents a coefficient that was not significant and was removed from the final model.

Table S4. Model fit information for the configural, metric and scalar models

	CFI	TLI	SRMR	RMSEA	Δ CFI	Δ TLI	Δ SRMR	Δ RMSEA
Configural	.981	.978	.084	.054				
Metric	.975	.973	.093	.060	-.006	-.005	.009	.006
Scalar	.954	.956	.092	.076	-.021	-.017	-.001	.016
Partial Scalar	.966	.967	.090	.066	-.009	-.006	-.003	.006

Table S5. Model parameters of the partial scalar model

	Factor loadings	Threshold 1	Threshold 2	Threshold 3
Poor neighborhood cohesion				
Item 1	1.00	-.46	.58	
Item 2	1.02	-.41	.67	
Item 3	0.76	-.29	.55	
Item 4	0.64	-.46	.19	
Item 5	0.56	-.54	.07	
Low intergenerational closure				
Item 6	1.00	-.04	.95	
Item 7	1.16	-.14	.73	
Item 8	0.93	-.10	.73	
Item 9	0.78	.12	.95	
Neighborhood disorder				
Item 10	1.00	-.03	.58	1.02
Item 11	1.07	.22	.68	1.12
Item 12	0.75	.12	.60	1.05
Item 13	0.78	.14	.55	.98
Social disorder				
Item 14	1.00	.12	.54	1.01
Item 15	0.88	-.12	.36	.86
Item 16	1.09	.12	.56	.94
Item 17	0.87	.00	.48	.92
Item 18	0.80	.17	.51	.85

Note. Factor loadings are unstandardized. The first loading was fixed to 1 for model identification. Thresholds are invariant thresholds.

Table S6. Factor means and variances as depicted by the partial scalar model

	Ghana	Jamaica	Pakistan	Philippines	Romania	South Africa	Sri Lanka	Vietnam
Means								
Poor neighborhood cohesion	.00	.72***	-.37	-.21	-.17	.10	-.28*	-.38***
Low intergenerational closure	.00	.12	.22	-.31	.22**	-.24	.26***	.26*
Neighborhood disorder	.00	.31*	.03	.51***	.13	.47***	.37*	-.06
Social disorder	.00	.42***	-.88***	.10	-.54**	.99***	.02	-.22
Variances								
Poor neighborhood cohesion	.76***	.56***	1.29***	.76***	.22***	.92***	.26***	.36***
Low intergenerational closure	.28***	25**	.52**	.52**	.21**	.65**	.13**	.25**
Neighborhood disorder	.79***	.49**	1.16**	.83***	.42***	.72***	.90***	.26**
Social disorder	.75***	.46**	.85**	.91***	.69**	1.38**	1.10***	.20**

Note. The means in group 'Ghana' were fixed to zero, as 'Ghana' was the reference group. The other mean values represent deviations from the mean of the reference group. Regarding means: *** p < .001; * p < .007 (i.e., the Bonferroni-corrected p-value of the standard p = .01 and p = .05 thresholds corresponding to 7 comparisons). Regarding variances: *** p < .001; ** p < .01. * p < .05.

Table S7. Correlation matrix for nomological networks in the 8 countries

Correlation matrix for nomological net in Ghana

		1.	2.	3.	4.	5.	6.	7.
1. Neighborhood cohesion	Pearson's r	-						
	p-value							
2. Intergenerational closure	Pearson's r	.58***	-					
	p-value	<.001						
3. Neighborhood disorder	Pearson's r	.10	.11	-				
	p-value	.22	.18					
4. Social disorder	Pearson's r	.22**	.10	.50***	-			
	p-value	.01	.26	<.001				
5. Depression	Pearson's r	.13	.04	.12	.15	-		
	p-value	.13	.68	.14	.07			
6. Stress	Pearson's r	.09	.06	.36***	.23**	.44***	-	
	p-value	.29	.51	<.001	.01	<.001		
7. Well-being	Pearson's r	-.23**	-.10	-.29***	-.28	-.43***	-.41***	-
	p-value	.01	.23	<.001	<.001	<.001	<.001	

Note. * p ≤.05, ** p ≤.01, *** p <.001

Correlation matrix for nomological net in Jamaica

		1.	2.	3.	4.	5.	6.	7.
1. Neighborhood cohesion	Pearson's r	-						
	p-value							
2. Intergenerational closure	Pearson's r	.49***	-					
	p-value	<.001						
3. Neighborhood disorder	Pearson's r	.19*	.15	-				
	p-value	.02	.07					
4. Social disorder	Pearson's r	.32***	.13	.56***	-			
	p-value	<.001	.11	<.001				
5. Depression	Pearson's r	.27***	.20**	.13	.22**	-		
	p-value	<.001	.01	.12	.01			
6. Stress	Pearson's r	.09	.06	.05	.18*	.59***	-	
	p-value	.25	.44	.52	.02	<.001		
7. Well-being	Pearson's r	-.22**	-.10	-.02	-.16*	-.52***	-.37***	-
	p-value	.01	.23	.78	.05	<.001	<.001	

Note. * $p \leq .05$, ** $p \leq .01$, *** $p < .001$

Correlation matrix for nomological net in Pakistan

		1.	2.	3.	4.	5.	6.	7.
1. Neighborhood cohesion	Pearson's r	-						
	p-value							
2. Intergenerational closure	Pearson's r	.56***	-					
	p-value	<.001						
3. Neighborhood disorder	Pearson's r	.00	.01	-				
	p-value	1.00	.88					
4. Social disorder	Pearson's r	.27***	.15	.43***	-			
	p-value	<.001	.08	<.001				
5. Depression	Pearson's r	.32***	.17*	.14	.24**	-		
	p-value	<.001	.04	.09	.00			
6. Stress	Pearson's r	.46***	.29***	.06	.21**	.66***	-	
	p-value	<.001	<.001	.47	.01	<.001		
7. Well-being	Pearson's r	-.49***	-.35***	.01	-.20**	-.61***	-.63***	-
	p-value	<.001	<.001	.91	.01	<.001	<.001	

Note. * $p \le .05$, ** $p \le .01$, *** $p < .001$

Correlation matrix for nomological net in the Philippines

		1.	2.	3.	4.	5.	6.	7.
1. Neighborhood cohesion	Pearson’s r	-						
	p-value							
2. Intergenerational closure	Pearson’s r	.47***	-					
	p-value	<.001						
3. Neighborhood disorder	Pearson’s r	.16*	.07	-				
	p-value	.05	.40					
4. Social disorder	Pearson’s r	.17*	.05	.76***	-			
	p-value	.03	.54	<.001				
5. Depression	Pearson’s r	.23**	.29***	.02	.01	-		
	p-value	.01	<.001	.84	.88			
6. Stress	Pearson’s r	.08	.18*	.09	.02	.50***	-	
	p-value	.30	.03	.29	.79	<.001		
7. Well-being	Pearson’s r	-.13	-.23**	-.13	-.07	-.43***	-.41***	-
	p-value	.11	.00	.12	.43	<.001	<.001	

Note. * p ≤.05, ** p ≤.01, *** p <.001

Correlation matrix for nomological net in Romania

		1.	2.	3.	4.	5.	6.	7.
1. Neighborhood cohesion	Pearson's r	-						
	p-value							
2. Intergenerational closure	Pearson's r	.63***	-					
	p-value	<.001						
3. Neighborhood disorder	Pearson's r	.20*	.05	-				
	p-value	.02	.53					
4. Social disorder	Pearson's r	.19*	.07	.70***	-			
	p-value	.02	.40	<.001				
5. Depression	Pearson's r	.20*	.09	.15	.07	-		
	p-value	.02	.29	.06	.38			
6. Stress	Pearson's r	.11	.06	.10	.08	.60***	-	
	p-value	.16	.45	.21	.33	<.001		
7. Well-being	Pearson's r	-.25**	-.14	.00	.09	-.66***	-.57***	-
	p-value	.00	.09	.98	.28	<.001	<.001	

Note. * p ≤.05, ** p ≤.01, *** p <.001

Correlation matrix for nomological net in South Africa

		1.	2.	3.	4.	5.	6.	7.
1. Neighborhood cohesion	Pearson's r	-						
	p-value							
2. Intergenerational closure	Pearson's r	.56***	-					
	p-value	<.001						
3. Neighborhood disorder	Pearson's r	.25**	.21**	-				
	p-value	.00	.01					
4. Social disorder	Pearson's r	.28***	.27***	.69***	-			
	p-value	<.001	<.001	<.001				
5. Depression	Pearson's r	.16	.13	.23**	.26**	-		
	p-value	.06	.11	.01	.00			
6. Stress	Pearson's r	.15	.12	.19*	.29***	.65***	-	
	p-value	.07	.15	.02	<.001	<.001		
7. Well-being	Pearson's r	-.14	-.06	.12	-.05	-.43***	-.42***	-
	p-value	.08	.48	.15	.57	<.001	<.001	

Note. * p ≤.05, ** p ≤.01, *** p <.001

Correlation matrix for nomological net in Sri Lanka

		1.	2.	3.	4.	5.	6.	7.
1. Neighborhood cohesion	Pearson’s r	-						
	p-value							
2. Intergenerational closure	Pearson’s r	.67***	-					
	p-value	<.001						
3. Neighborhood disorder	Pearson’s r	.10	.18*	-				
	p-value	.23	.02					
4. Social disorder	Pearson’s r	.08	.19*	.83***	-			
	p-value	.34	.02	<.001				
5. Depression	Pearson’s r	.04	.06	.08	.14	-		
	p-value	.59	.44	.34	.10			
6. Stress	Pearson’s r	.10	.16*	-.02	.10	.63***	-	
	p-value	.23	.05	.85	.24	<.001		
7. Well-being	Pearson’s r	-.18*	-.22**	-.10	-.15	-.50***	-.40***	-
	p-value	.02	.01	.22	.07	<.001	<.001	

Note. * p ≤.05, ** p ≤.01, *** p <.001

Correlation matrix for nomological net in Vietnam

		1.	2.	3.	4.	5.	6.	7.
1. Neighborhood cohesion	Pearson's r	-						
	p-value							
2. Intergenerational closure	Pearson's r	.72***	-					
	p-value	<.001						
3. Neighborhood disorder	Pearson's r	.24**	.14	-				
	p-value	.00	.10					
4. Social disorder	Pearson's r	.33***	.32***	.49***	-			
	p-value	<.001	<.001	<.001				
5. Depression	Pearson's r	.19*	.23**	.20**	.40***	-		
	p-value	.02	.00	.01	<.001			
6. Stress	Pearson's r	.14	.23**	.11	.27***	.51***	-	
	p-value	.09	.01	.17	<.001	<.001		
7. Well-being	Pearson's r	-.16*	-.26**	-.12	-.16*	-.49***	-.48***	-
	p-value	.05	.00	.13	.05	<.001	<.001	

Note. * $p \leq .05$, ** $p \leq .01$, *** $p < .001$
